# Safety aspects of atmospheric pressure helium plasma jet operation on skin: *In vivo* study on mouse skin

**DOI:** 10.1371/journal.pone.0174966

**Published:** 2017-04-05

**Authors:** Spela Kos, Tanja Blagus, Maja Cemazar, Gregor Filipic, Gregor Sersa, Uros Cvelbar

**Affiliations:** 1 Department of Experimental Oncology, Institute of Oncology Ljubljana, Ljubljana, Slovenia; 2 Faculty of Health Sciences, University of Primorska, Izola, Slovenia; 3 Jozef Stefan Institute, Ljubljana, Slovenia; Universite Toulouse III Paul Sabatier, FRANCE

## Abstract

Biomedical applications of plasma require its efficacy for specific purposes and equally importantly its safety. Herein the safety aspects of cold plasma created with simple atmospheric pressure plasma jet produced with helium gas and electrode discharge are evaluated in skin damage on mouse, at different duration of exposure and gas flow rates. The extent of skin damage and treatments are systematically evaluated using stereomicroscope, labelling with fluorescent dyes, histology, infrared imaging and optical emission spectroscopy. The analyses reveal early and late skin damages as a consequence of plasma treatment, and are attributed to direct and indirect effects of plasma. The results indicate that direct skin damage progresses with longer treatment time and increasing gas flow rates which reflect changes in plasma properties. With increasing flow rates, the temperature on treated skin grows and the RONS formation rises. The direct effects were plasma treatment dependent, whereas the disclosed late—secondary effects were more independent on discharge parameters and related to diffusion of RONS species. Thermal effects and skin heating are related to plasma-coupling properties and are separated from the effects of other RONS. It is demonstrated that cumulative topical treatment with helium plasma jet could lead to skin damage. How these damages can be mitigated is discussed in order to provide guidance, when using atmospheric pressure plasma jets for skin treatments.

## Introduction

Gaseous plasma treatment at atmospheric pressure has developed in the last few years into innovative and growing field of research with specific emphasis in biomedical applications. Current developments relate to the design of specialized plasma sources and delivery techniques, to maintain prescribed safety standards and to investigate the processes that are of relevance in medicine and health care [1]. Clinical potential of non-thermal plasmas has widened to sterilization of inert surfaces [2–5] and living tissues [6], inducing blood coagulation [7], stimulation of cell proliferation and enhancement of cell transfection [8]. Up to now, clinical research in plasma medicine has been mainly focused on application in dermatology and aesthetic surgery for the purposes of tissue regeneration in order to improve healing of infected or chronic wounds as well as to treat infective and inflamed skin diseases [9–13]. Since topical treatment is one of the leading medical approaches for plasma applications, skin damage caused by non-thermal plasma needs to be carefully evaluated [9, 14].

Although plasma treatment at atmospheric pressures is considered as a safe and harmless approach for the skin, it could result in noticeable skin malformations [15]. Plasma is typically generated by a discharge which causes ionization, excitation or dissociation of gas molecules leading to creation of various gaseous plasmas. This is done in gases such as argon, helium, oxygen, nitrogen or mixtures thereof. Sequently, ionized or excited gaseous molecules, atoms and even photons interact with the target tissue which results in generation of surface reactive species with biological potential, thermal heating, molecule scission or even creation of secondary species/photons [16]. Consequently, these species or better said plasma-surface interactions cause damage at observed treated skin tissue. These damages caused are thermal injuries, UV radiation damages or results from the generation of reactive gaseous species, such as oxygen (ROS), nitrogen (RNS) or jointly named RONS [16, 17]. Furthermore, skin damage highly depends on different types of plasmas used and a variety of other parameters such as a skin structure, the dosage of the plasma species, the exposure time to the treatment [15], etc. Hence, it is also essential to identify the boundaries of plasma toxicity to the skin after the treatment.

The aim of this study was to evaluate the extent of skin damage after the atmospheric pressure helium plasma jet treatment using different flow-rates and exposure times to plasma. Mouse skin was selected as a model to evaluate possible skin damage caused by application of frequently used atmospheric pressure plasma jet (APPJ). The direct and indirect damage was monitored and evaluated using surface analysis techniques as well as plasma characterization. For surface analyses, optical microscopy, molecule labelling, histology and IR imaging were used, whereas plasma was characterized by optical emission spectroscopy. The direct skin effect of plasma treatment caused by thermal heating was determined by IR imaging at the treated site. The skin and plasma were viewed also by optical emission spectroscopy during the treatment to try to find some correlations with generated plasma and skin damage. Moreover, given study provides also the evidence on toxic effects of plasma on treated skin and sets the boundaries of its toxicity by evaluating different plasma parameters. The optimized parameters of plasma flow rate and treatment time set the stage for further experiments and translation of plasma treatment for the purpose of different clinical applications, such as wound healing, skin cancer treatment or intradermal gene delivery of plasmids in gene therapy.

## Materials and methods

### Reagents

Four kDa fluorescein-isothyocianate (FITC) labelled dextran (Sigma-Aldrich, St. Louis, MO, USA) was used as a marker for direct skin damage caused by plasma treatment. After resuspension of FITC in phosphate buffered saline (PBS), FITC was washed twice for 2 h through 2 kDa ultrafiltration spin column (Vivaspin, Sartorius Stedim Biotech, Goettingen, GE) in order to remove free FITC or low-molecular weight contaminants. The rest of components with high molecular weight were resuspended in PBS to a final concentration of 37.5 mg/mL. Until the usage, FITC labelled dextran was stored in the dark at 4°C.

### Animals and skin preparation

All procedures were performed in compliance with the guidelines for animal experiments of the EU directive (2010/63/EU) and the permission from the Veterinary Administration of the Ministry of Agriculture, Forestry and Food of the Republic of Slovenia (permission no. 34401-4/2012/4). In order to evaluate skin damage 8–12 week old female Balb/c mice (Envigo, Udine, IT) were used. All mice were housed in pathogen-free condition with 12-hour light cycles and provided food and water *ad libitum*. For each experimental condition 3–5 mice were assigned. A day before plasma treatment mice were shaved and depilated on the left flank and remaining hair was additionally removed with depilatory cream (Veet® Sensitive Skin, Reckitt Benckiser, UK). Treated mice were humanely sacrificed by cervical dislocation or inhalation of CO_2_.

### Plasma system

The plasma system consisted of hand-held atmospheric pressure plasma jet (APPJ) operating with electrode discharge in helium gas and connected power supply. As an electrode, a copper wire with diameter of 0.1 mm was inserted inside 110 mm long and 1.2 mm inner diameter borosilicate glass tube. The electrode extended almost to the end of the tube, stopping at about 1 mm before the glass tube orifice. Electrode was connected to a commercial 25 kHz high voltage alternating current power supply (3 kV, 2 mA) supplied from Conrad Electronic. The ground electrode was not used in the experiment. Helium of purity 5.0 (Messer Slovenija, Ruse, SI) was leaked into glass tube through Bronkhorst Mass-view flow controller at different flow rates. The optical emission spectroscope Avantes AvaSpec 3648 with a nominal spectral resolution of 0.8 nm in the range from 200 to 1100 nm was used for plasma characterization during the measurements.

### Plasma treatment

All animals were initially anesthetized with inhalation anaesthesia in the induction chamber using 2% isoflurane (Nicholas Piramal India, London, UK) mixture with oxygen. They were placed on a flat surface, with their snout in the inhalation tube. Treatments were carried out under constant supply of 2% isoflurane in oxygen delivered through the inhalation tube. Characterization of plasma system was previously described elsewhere [18, 19]. In this study, mice were treated with helium plasma with a 2 cm space between the end of the jet orifice and the target tissue of mouse left flank. The considerable distance of 2 cm was set to reduce the influence on plasma properties caused by slight mouse movements during breathing. Plasma jet was directed perpendicularly to the treated skin tissue. However, constant mouse movements during the experiment could shift the angle of plasma jet, and this could lead to smaller angle deviations in plasma jet orientation. Plasma conditions were assigned for each experimental group, with varying delivery time and plasma flow rate. 5 groups were treated with applied flow rate 5 L/min and the delivery time either 0.5, 1, 2, 3, or 4 min. In the case of 4 min treatment time, skin damage was additionally evaluated using plasma flow rates ranging from 1–5 L/min. Immediately after plasma treatment, a patch soaked with 100 μL of FITC labelled dextran was applied on the treated skin tissue of live mouse. After 1 h, the patch was removed and the fluorescence imaging was employed *in vivo*. Optical emission spectrum was collected with an optical fibre connected to lens 10 mm away from the mouse and in the line of plasma-skin interaction zone.

### Fluorescence microscopy and data analysis

Fluorescence microscopy was carried out with a Zeiss Stereo Lumar.V12 (Zeiss, Jena, GE) fluorescence stereomicroscope equipped with an MRc.5 digital camera (Zeiss). Before the procedure mice were anesthetized with inhalation anaesthesia in induction chamber using 2% isoflurane in oxygen and remained anesthetized during the experiment to prevent any larger movements during the observation. At each time point, images were taken under fluorescent light (RGB images) or under visible light (VIS images). The first image was taken 1 h post-treatment, the rest followed at 3, 24, and 48 h post- treatment. The area of skin damage was analysed with the image analysis software AxioVision (Zeiss). The indirect damage was quantified from RGB and VIS images, the total damage was quantified from VIS images. The percentage of indirect damage was calculated from the total and indirect skin damage.

### Histology

Histology analysis was performed for the plasma conditions at 1, 2, 3, 4, 5 L/min and delivery time 4 min. After the treatment, patch soaked with FITC labelled dextran was applied on the tissue as described before. To determine the depth and extent of skin damage the region of the skin exposed to the treatment was excised 3 h post-treatment. The excised skin was fixed in Zn fixative (BD Pharmingen^TM^, BD Bioscience, San Diego, CA) for 24 h and then stored in 70% ethanol until embedding in paraffin. 5 μm thick sections were cut and stained with haematoxylin and eosin or Masson’s trichrome. The slides were observed with BX-51 microscope (Olympus, Hamburg, GE) equipped with a digital camera DP72 (Olympus).

### IR imaging

The potential skin effects due to thermal heating during plasma-surface interaction were monitored by thermal imaging using IR camera FLIR SC5000. The images recorded the surface of mouse skin with plasma jet and the zone of interaction. The thermal images detected were adjusted for 0.92 emissivity of human skin, where the background temperature was adjusted to room temperature (23°C). The potential results presented can deviate for a maximum of a couple of degrees.

### Statistical analysis

For statistical analysis Sigma Plot software (Systat software, London, UK) was used. Mean and standard errors were calculated for each treatment group. Comparison between groups was done by using one-way analysis of variance (ANOVA) followed by Holm-Sidak test. A value p<0.05 was considered statistically significant. The values were expressed as arithmetic mean (AM) ± standard error of the mean (SEM).

## Results

### Early and late skin damage

**Fig 1:**
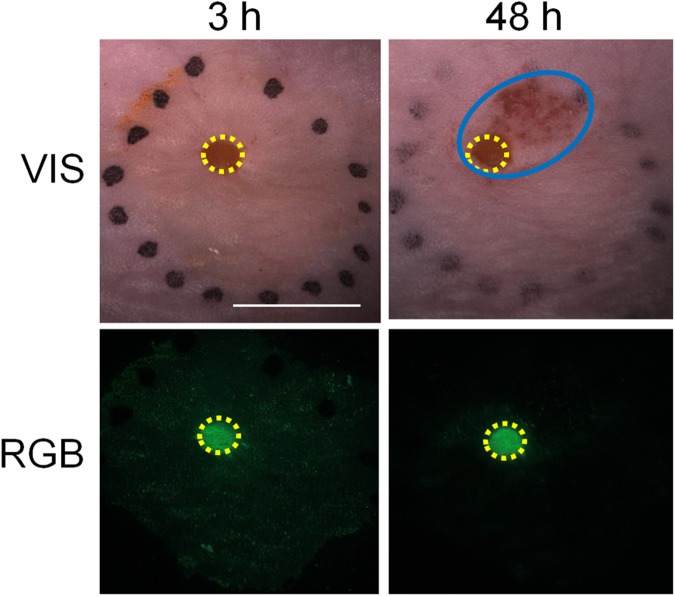
Microscope images of the direct and indirect skin damage caused by plasma jet treatment. Images were taken under stereomicroscope at 3 h and 48 h post-treatment. The direct and indirect damage can be observed under visible light (VIS). FITC labelled dextran was observed under RGB conditions and was ascribed as an indicator of direct skin damage. Yellow circles represent the direct skin damage; the indirect damage is labelled in blue. *Scale bar*: 5 mm.

*Early skin effects* were observed immediately after the plasma jet treatment. The skin burns were noticed at the site where the plasma jet was in the contact with the mouse skin. This injured area of the skin, observed already during the treatment, was considered as a *direct skin damage*. To quantitatively evaluate these changes, FITC labelled dextran was used as the indicator of direct skin damage. Namely, fluorescently labelled dextran, applied topically to the treated area, indicated the area of the skin that was affected by direct plasma damage. Surprisingly, after 24–48 h the additional damage around the direct plasma damage was observed. This damage was presented as oedema around the treated area, and was not subjected to initial direct plasma damage (Fig 1). For this reason, it was defined as *late effect* and presented *indirect skin damage*. FITC did not progress into the area of indirect damage, indicating that indirect skin damage evolved later than the direct skin damage. This helped separating primary and secondary damage to the skin caused by the plasma treatment.

### Relating plasma treatment time with skin damage

**Fig 2:**
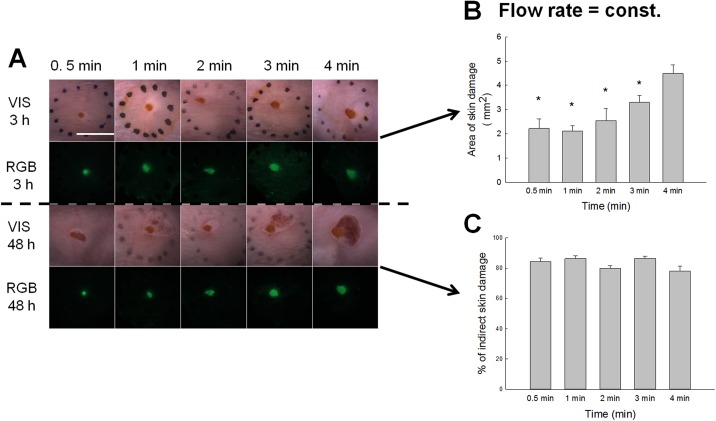
Skin damage observed under different treatment times at constant gas flow rate of 5 L/min. A) Representative images of the skin damage in the treated region. Treated skin regions were observed under stereomicroscope. Images were taken under visible light (VIS) and the fluorescence was observed under RGB (red, green, blue) parameters. Scale bar: 5 mm. B) The area of the direct skin damage was measured 3 h post-treatment and statistically evaluated from RGB images. Error bars indicate SEM. *P value < 0.05 between the selected group and the group exposed to the longest treatment time (treated 4 min under 5 L/min). C) Percentage of indirect skin damage quantified from the total damage (direct and indirect), measured 48 h post-treatment. Damage was statistically evaluated from RGB image. Error bars indicate SEM.

The simplest question arising during plasma jet treatment of skin is how treatment time can influence the direct skin damage. For this reason, the area of direct skin damage was measured after different treatment times ranging from 0.5 s to 4 min, whereas helium (He) gas flow rate was kept constant for all time intervals (Fig 2A). The results as presented in Fig 2B indicated the direct correlation between the area of direct skin damage and increased treatment time. The highest damage achieved was detected at longest plasma treatment time of 4 min which resulted in severe skin burns. On contrary, the indirect skin damage was not dependent on treatment time. These changes were observed after the treatment within 24 to 48 h, where surface area of damage varied randomly to treatment time (Fig 2C). The damage area was more dependent on the angle of plasma jet stream. When the stream was perpendicularly oriented to the skin, indirect skin damage was observed, compared to when the stream was not completely perpendicular to the skin. The direct and indirect skin damage could be observed up to 2 weeks post–treatment. Past this, the wound was healed due to the fast regeneration of mouse skin.

### Influence of gas flow rate and the extent of direct skin damage

**Fig 3:**
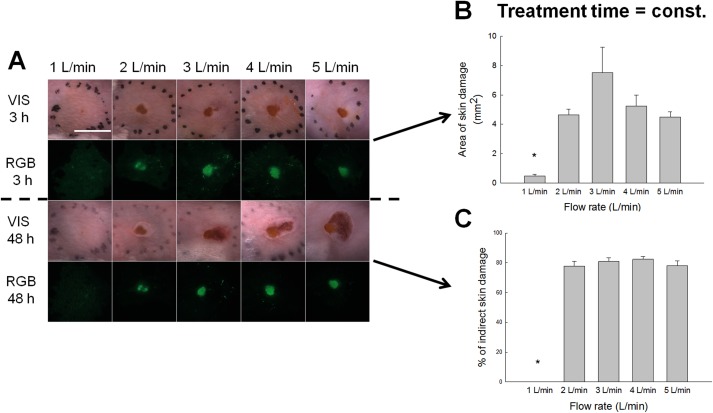
Skin damage observed under different plasma flow rates. A) Representative images of the skin damage in the treated region. Treated skin regions were observed under stereomicroscope. Images were taken under visible light (VIS) and the fluorescence was observed under RGB (red, green, blue) parameters. Scale bar: 5 mm. B) The area of the direct skin damage was evaluated from RGB images. Error bars indicate SEM. *P value < 0.05 between the selected group and the group treated under the highest plasma flow rate (treated 4 min under 5 L/min). C) Percentage of indirect skin damage calculated from the total damage (direct and indirect) measured 48 h post-treatment. Damage was statistically evaluated from RGB image. Error bars indicate SEM.

First experiments exhibited variations in observed skin damage, when different He gas flow rates were used in plasma jet. Namely, the flow rate can bring reactive species further away from the end tip of the tube (tube orifice) where plasma is generated to the surface. Moreover, increased flow rates can also increase or shorten the plasma jet length as well as bring plasma from almost laminar to more turbulent flow state [18–22]. Similarly as the jet flow is interacting with the surface, gaseous reactive species are spread over surface [22]. Additional influence can also come from so-called coupling of jet with the surface, where streamers are formed and higher currents are driven onto the skin. For this reason, the area of the direct skin damage was measured after the plasma treatment under different gas flow rates ranging from 1 to 5 L/min (Fig 3A). Under 1 L/min, the gas flow was weak and barely reached the skin surface, whereas no streamers were observed. Consequently, the skin burns were minimal. Under higher flow rates, the damage was more extensive (Fig 3B). Meaning, the plasma treatment was most aggressive at flow rate 3 L/min, where direct burns ranged to about 8 mm^2^ as evaluated from RGB images. The visible damages were even more extensive, but were subjected to geometric jet-skin interactions as mentioned before. Except from the flow rate of 1 L/min, the indirect damage was observed under all other flow rates (Fig 3C). This was connected to gas flow dynamics, plasma jet properties and coupling.

**Fig 4:**
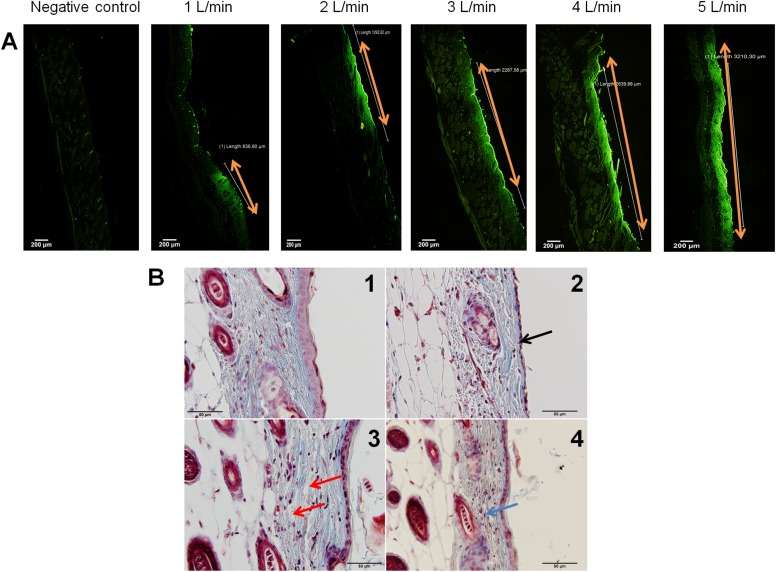
Histological evaluation of skin damage. Skin treated under different plasma flow rates (from 1 to 5 L/min) was excised and 5 μm thick sections were cut and stained with haematoxylin and eosin or Masson’s trichrome. Skin sections were observed under BX-51 microscope (Olympus) and histologically evaluated. A) The skin exposed to FITC, visualized under RGB conditions. Skin sections were stained with hematoxylin and eosin. Orange arrow marks the central fluorescent area, where the skin samples were further histologically evaluated. *Scale bar*: 200 μm. B) Histological evaluation of damaged skin, performed under visible light. Skin sections were stained with Masson’s trichrome. 1) Control un-treated skin. 2) The black arrow points to reduced cell layers of epidermis (single layer of epidermal cells visible under flow rate of 5 L/min).3) Red arrow presents the oedema and destructed structure of collagen fibers (obtained under flow rate of 4 L/min). 4) Blue arrow presents the immune cell infiltration (obtained under flow rate of 3 L/min). *Scale bar*: 50 μm.

The stereomicroscope evaluations revealed only a part of the story of the skin damage which could be observed from the topical observations. In order to precisely determinate skin damage in deeper layers, the treated areas of the skin were histologically evaluated. To determine the damaged skin area, the skin sections were firstly observed by fluorescent microscope. At the area where the fluorescent signal of FITC (Fig 4A) was detected, histological images were taken (Fig 4B). Histological evaluation reconfirmed the macroscopical observations of skin wounds under different flow rate parameters. Compared to the control un-treated skin section (Fig 4B1), under 1 L/min there was no damage observed in excised skin samples. The dermis as well as epidermis were intact. With higher flow rates, the damage of epidermis progressed, noticed as the cytoplasmic eosinophilia in epidermal cells (red stained epidermal cells), reduced cell layers of epidermis (Fig 4B2) and the cells with flat condensed nuclei (picnotic cell nuclei) (Fig 4B2). Skin damage further progressed to damage of the dermis, noticed as the oedema (accumulation of the fluid in the interstitium) (Fig 4B3) with signs of inflammation (infiltration of immune cells) (Fig 4B4), when the flow rate of 5 L/min was applied. Numerous hair follicles were observed in excised skin sections, which remained undamaged after the plasma treatment.

### Skin damage from temperature and RONS at the treatment site

**Fig 5:**
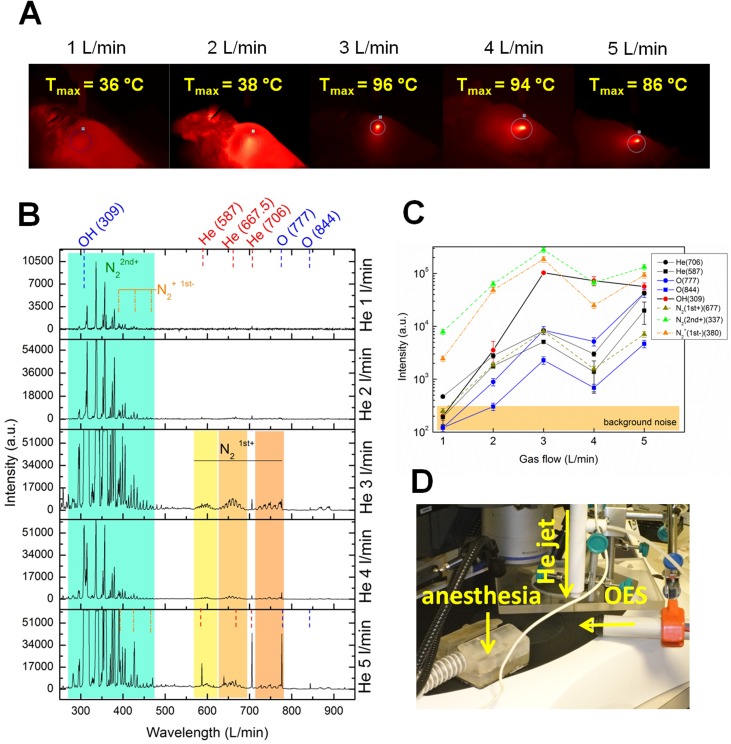
The effect of temperature and gaseous RONS formation on skin damage. A) The surface temperature was measured on the mouse skin during the plasma treatment using IR camera. Mice were treated under different flow rate conditions. The maximum temperature caused by plasma treatment is indicated on each image. B) OES spectrum of RONS generated in plasma flow during the skin treatment at the treatment site. C) Intensity of different RONS, measured under different flow rate parameters. D) Combined experimental setup for the purposes of OES measurements. The mice were placed under helium (He) jet and were anesthetized during the procedure. The spectrometer was placed perpendicular to He plasma jet, directed to the treated skin area.

In order to separate the observed thermal skin damage from the effects of plasma reactive gaseous species like reactive oxygen and nitrogen species (RONS), the IR (Fig 5A) and OES measurements were used (Fig 5B–5D), respectively. Using the IR camera, the averaging temperature within 10 ms was measured on the mouse skin during the plasma treatment under different flow rate conditions. When plasma jet interacted with the surface it was positioned at the same area spot, so the thermal effect was monitored as cumulative over the total treatment period. For this reason, the highest temperatures were achieved at the end of treatment time with maximum temperature spots as presented in Fig 5A. The surface temperatures normally raised quickly and then saturated around the maximal presented temperatures. Under the smallest flow rate 1 L/min, the temperature changes on the skin caused by the plasma application were almost not detected. The skin temperatures were slightly elevated at plasma flow rate 2 L/min. Under higher plasma flow rate conditions (3–5 L/min), significantly elevated temperature was measured and reached up to 96°C on the treated mouse skin. The optical emission spectroscopy provided the evidences of intensity of a wide range of gaseous RONS formed in the plasma gas flow, when the mouse skin was treated under different flow rate conditions. When plasma jet created in pure He expands in open air and reaches the surface, a number of secondary species are created at the interacting surface layer or in reactions with surrounding air. The part of plasma species which emit photons can be detected by OES and give some idea on RONS on the surface which might influence the skin damage (Fig 5B). The main observed peaks represented are helium (He 587, 667.5 and 706 nm), hydroxyl molecules (OH 309 nm), and atomic oxygen (O 777 and 844 nm) as well as nitrogen reactive species from molecular bands. The raised nitrogen bands correspond to molecular ion N_2_^+^ first negative system (B^2^Σ_u_^+^—X^2^Σ_g_^+^) or excited N_2_ second positive system (B^3^Πu—B^3^Π_g_) and N_2_ first positive system (B^3^Πu−A^3^Σ_g_^+^). For presentation of these systems intensities, bands at 380 nm, 667 nm, and 337 nm were used, respectively. The highest optical emission intensity of RONS was detected under flow rate at 3 and 5 L/min, whereas the most significant and intensive was under 3 L/min (Fig 5C). This spectrum is characterized by dominant intensity of nitrogen bands and other peaks like helium which follow the same trend. As observed, the highest intensity of plasma is achieved under 3 L/min flow rate. Later the intensities slightly drop as a consequence of mode moving to more turbulent flow and mechanisms of forming other species in plasma. Compared to 3 L/min, less RNS were formed under 5 L/min with higher intensity of ROS. However a striking difference is the behaviour of OH molecules which do not follow the same trend as other the spectral lines. Moreover, their intensities and trend seem to correspond to maxima of skin heating. This indicates that surface heating is connected to intensity emission from OH molecules, where OH molecules are probably result of some surface thermal reactions or even participate in them.

## Discussion

Atmospheric pressure plasma treatments of skin are generally considered a safe approach, although some reports already indicated that tissues can be damaged after a certain dosage of plasma radicals [23, 24]. However, primary or early effects (direct) and secondary or long-term effects (indirect) are not well known. In contrary to most reports where evaluation of plasma safety aspect is only leaned on tissue cells like human keratinocytes HaCaT, which are used as *in vitro* model of skin [25], this paper sheds more light on *in vivo* treating mice. Herein the single-point area cumulative treatment is considered, as well as geometrical orientation and post-treatment effects in respect to used plasma conditions with different gas flow rates and treatment times at a constant distance. Moreover, the presented study is to point out that plasma is not that safe as presented and can cause tissue damage. Herein it is also clearly demonstrated that the extent of the tissue damage depends on different APPJ discharge parameters resulting in different plasma properties and coupling, where streamers are formed between electrode in orifice and mouse skin. Here it is worth noticing that the used source is frequently used as APPJ, but not necessary present a typical plasma medical device developed by other research groups, where some negative plasma properties are omitted in advance. Hence, it is important to understand how to optimize the simple operating parameters in practice going back to initial designs of APPJs and finding the balance between the optimal efficiency of plasma treatment and maximal safety of the procedure for patients treatment.

When comparing different studies and the effect of plasma on the treated tissue, it needs to be considered that experimental setups vary between the studies, with different plasma sources and different cellular and tissue system used to study the plasma effects [16]. All plasma parameters, as the results of operating discharge parameters, differently contribute to the safety of the treatment and need to be evaluated before the translation of the plasma treatment into the medical practice and treatment of patients. Our results clearly indicate that skin damage progresses with prolonged treatment time and increasing gas flow rates. With increasing flow rates the temperature on treated skin rises and the gaseous RONS formation increases. This results in direct skin damage, observed as a skin burn. As it is evident from the histological results, the most extensive area of direct skin damage was observed at flow rate of 3 L/min. Moreover, at 3 L/min the maximum temperature rise on treated skin was measured as well. Despite this, the deepest skin malformation with complete damage of epidermis and deeper situated dermis was observed at 5 L/min, where the maximum peak of oxidative species was detected. The oxidative species arise either from the surrounding air or surface interactions, especially as a result of streamer formation with higher flow rates, larger than 2 L/min, which also corresponds to extensive skin heating. Additionally, oxidative species are known for their rapid reaction with biological compounds and oxidative reaction with treated tissues [26], resulting in more severe extent of skin damage. It seems that cumulative effect streamer formation and more ROS species created in plasma–skin interaction zone, as measured from atomic oxygen O and hydroxyl OH radicals, correspond to higher extent of skin damage. Here it is worth noticing that there are probably other ROS species like metastabels at the surface, which cannot be detected with OES and more sophisticated methods of plasma diagnostics would be needed. Indication on role of gaseous ROS species is given through monitoring intensity of hydroxyl spectral line, which is almost identical to maximum reached skin surface temperature. Both follow the same trend and maximum of OH intensity corresponds to maximum of 96°C skin temperature reached at 3 L/min. Therefore, the major source of skin heating could be observed through OH radicals, whereas combination of surface heat released could be through resistive heating and surface chemistry, due to streamer and RONS formation which both contribute to the direct skin damage after the plasma treatment. Beside this, higher surface temperature of skin also contributes to higher evaporation effect. Namely, the surface wetness is strongly correlated to OH density distribution [27]. Moreover, it was correlated that OH density has maximum at the center of the plasma jet impinging onto surface. Same authors also observed that beside humidity, the densities of OH and O radicals are well connected to the helium flow rate. As in presented study, the highest OH densities were observed for 3 L/min flow rates, lower for 6 L/min and the lowest for 1.5 L/min He flow rate, whereas maximum OH densities were achieved between 1 and 2 cm distance from jet orifice (for 3 and 6 L/min). These observations correlate well to measurements in this study and extend of direct damages, as well as temperature distribution function on surface.

Additionally, this study presents the direct and indirect skin damage caused by plasma treatment, which were to the best of our knowledge not reported before. As was described above, direct skin damage was detected immediately after the treatment, and was described as skin burns on the area where the plasma came into contact with the skin. These skin burns could be simply labelled and monitored with FITC labelled dextran, and were attributed to the released heat and RONS effects. Contrary to the direct damage, the indirect skin damage was observed only 48 h after the treatment, noticeable as an oedema around the treated area. The area of oedema was not penetrated by FITC labelled dextran. It was noticed that the indirect malformations could appear when the plasma jet was not at the direct perpendicular position with the treated tissue, but oriented under an angle, smaller than 90°. Since the constant mouse movementduring the experiment shift of the angle of interacting jet was rather frequent phenomena. The simulations show that species created at interface like OH (as well as *in-situ* formed O radicals) also enable creation of pores in membranes leading to degradation of skin barrier function, enabling penetration of other radicals and molecules into skin tissue [28]. However, it revealed an interesting proof of damage of surrounded tissue, which is probably caused by RONS produced at the plasma-surface interphase. These RONS do not originate from plasma gas phase, but are products at plasma-skin or plasma-biofluid interactions and they diffuse under the skin only after the treatment. This occurs after surface layer is damaged and skin barrier function disabled. The creation of these surface-liquid RONS is well known from plasma-liquid interactions, where various solvated species are created, such as hydrogen peroxides (H_2_O_2_), superoxide anions (O_2_^-^), hydrated electrons, nitrates, nitrides, etc. [29–32]. Also the role of intercellular ROS generated in the skin tissue as a result of plasma treatment and their involvement in skin damage extension should not be overlooked [33]. As a result of diffusion of these species, the surrounding tissue is inflamed. However, more research is needed to unravel these very complex phenomena occurring in lower layers of the skin and pinpointing responsible RONS species. Despite this, the major conclusion is that the indirect skin damage does not depend on treatment time nor plasma flow rate conditions.

Beside the experimental setups and gas used for the reaction, attention should be paid also to the plasma source. During recent years a wide range of different plasma sources dedicated for biomedical applications has been reported [34]. Generally, three types of plasma sources are applicable for clinics; barrier discharges, corona discharges and plasma jets [35–38]. In this study, plasma jet was used and evaluated for its effect on the skin. Plasma jets are popular devices that blow plasma out of the device, overcoming the disadvantage of conventional plasmas confined between electrodes [39]. The length of the plasma plume generated is up to a few centimeters in the air, so plasma jets are suitable for direct treatment of medical targets. Furthermore, plasma jet is localized and contracted plasma allowing primarily a spot-like treatment. The advantage of spot-like plasma source is that the treated area could be exactly localized and the treatment can be limited to the target area, without affecting the surrounded tissue that should not be treated [16]. However, this is not entirely true, as demonstrated in our study. Even the surrounding, non-treated tissue could be affected, as was evident from the indirect skin damage. Furthermore, the drawback of spot-like treatment, as was presented in our study, is the tissue damage caused by continuous and cumulative treatment limited to small skin area. It resulted as skin burn. We assume that the direct skin damage would be less severe, if the same incoming plasma particle flux would be distributed around a bigger area. It could be achieved by gradually moving of the jet over the surface or with other kinds of plasma sources, such as dielectric barrier discharge, allowing larger area treatment [16]. Therefore, with further advances and improvements of plasma sources the tissue damage could be ameliorated.

It is important to select an appropriate plasma system suitable for a particular application. The given example is the ROS production. Although ROS described in this paper are the negative factor involved in the progress of direct and indirect skin damage, for some of medical application their extensive production is even welcome [40]. ROS production caused by non-thermal plasma appeared as a promising approach for the treatment of various diseases including cancer. Non-thermal plasma generated high amount of ROS, leading to the formation of DNA damage [41]. It resulted in cell cycle arrest and consequently in apoptosis induction [42]. This strategy is particularly promising in the context of tumor treatment [42], as was already described on melanoma tumor model [33]. Similar conclusions were made, observing the role of ROS in the wound healing process [43]. ROS in smaller amount play a crucial role in the orchestration of the normal wound healing response, but could in higher dosage lead to extensive skin damage, as presented in this study.

In the future, it will become a general requirement to adapt special plasma sources to specific medical applications and variable target tissues. Such adaptability is also needed regarding plasma parameters, such as plasma flow rate and treatment time, to realize different performance characteristics during medical plasma treatment process. Therefore, a lot of basic research is needed to get more insight into detailed mechanisms of plasma-induced effects on living tissues and the particular role of each plasma component [16].

## Conclusion

To conclude, this study points out that the use of atmospheric pressure plasma jet is not as safe as presented by many authors, and can cause tissue damage, but some precautions should be considered when using different atmospheric plasma jets. The extent of the tissue damage depends on different APPJ discharge parameters resulting in different plasma properties and coupling. All plasma parameters as results of operating discharge parameters differently contribute to the safety of the treatment and need to be evaluated before the translation of the plasma treatment into the medical practice and treatment of patients. The results of this study clearly indicate that skin damage progresses with prolonged treatment time and increasing gas flow rates. With increasing flow rates, the temperature on treated skin grows and the RONS formation as well as streamer formation rises. This results in direct and indirect skin damage. The direct damage was described as skin burns, which could be simply labelled and monitored with FITC, and were attributed to the released heat and RONS effects. Contrary to the direct damage, the indirect skin damage was observed only after 24 to 48 h after the treatment, noticeable as an oedema around the treated area. The damage of surrounded tissue was probably caused by RONS produced at the plasma-surface interphase. The indirect skin damage does not depend on treatment time nor plasma flow rate conditions. The study opens the possibilities for further improvements of topical plasma treatment, with the tendency to minimize the tissue damage. This would improve the efficiency and safety of the APPJ procedure and promote its translation into the clinics.
